# A simple technique using a Venflon to fix fractures of the glenoid

**DOI:** 10.1308/rcsann.2023.0066

**Published:** 2023-12-01

**Authors:** A Kapasi, C Uzoigwe, D Barlow, A McMurtrie

**Affiliations:** ^1^ Wrexham Maelor Hospital, Betsi Cadwaladr University Health Board, UK; ^2^ Harcourt House, Sheffield, UK

## Background

Glenoid fractures account for 10%–48% of scapular fractures.^1^ Fractures representing more than 25% of the articular surface are associated with shoulder instability. Surgical fixation is indicated in these circumstances.^1,2^ This can be challenging given the glenoid size, its relation to the humeral head and the proximity of neurovascular structures.

By necessity, fixation instrumentation is of small calibre. The surrounding soft tissues can impede achieving the optimum trajectory of fixation devices. This is compounded by the orientation of the glenoid and the position of fracture fragments.^3^ We describe a technique for facilitating the positioning of screws during glenoid fixation.

## Technique

**Figure 1:**
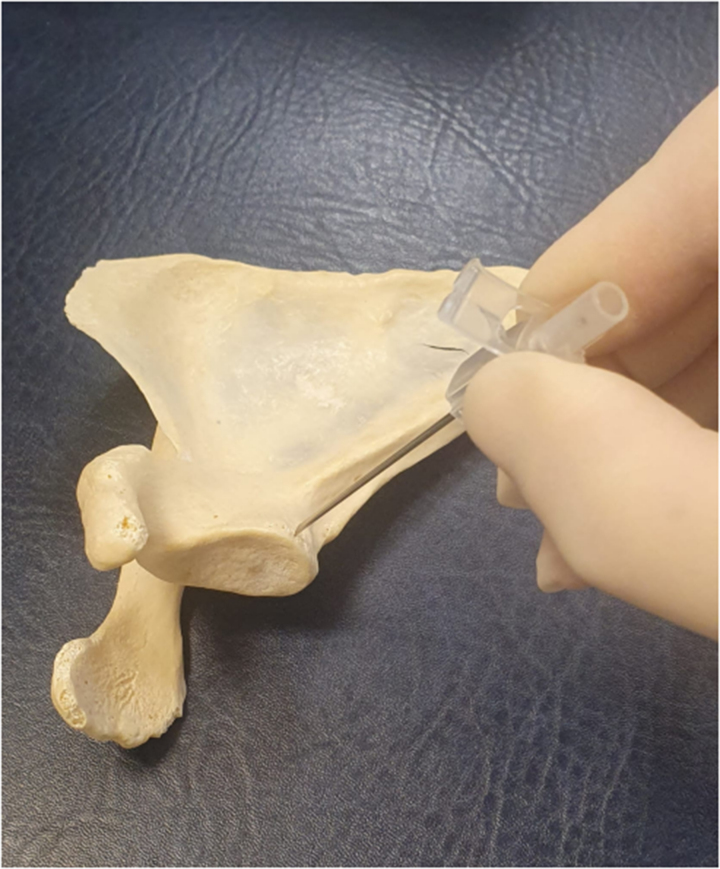
Orange cannula with sleeve removed positioned against glenoid rim

**Figure 2:**
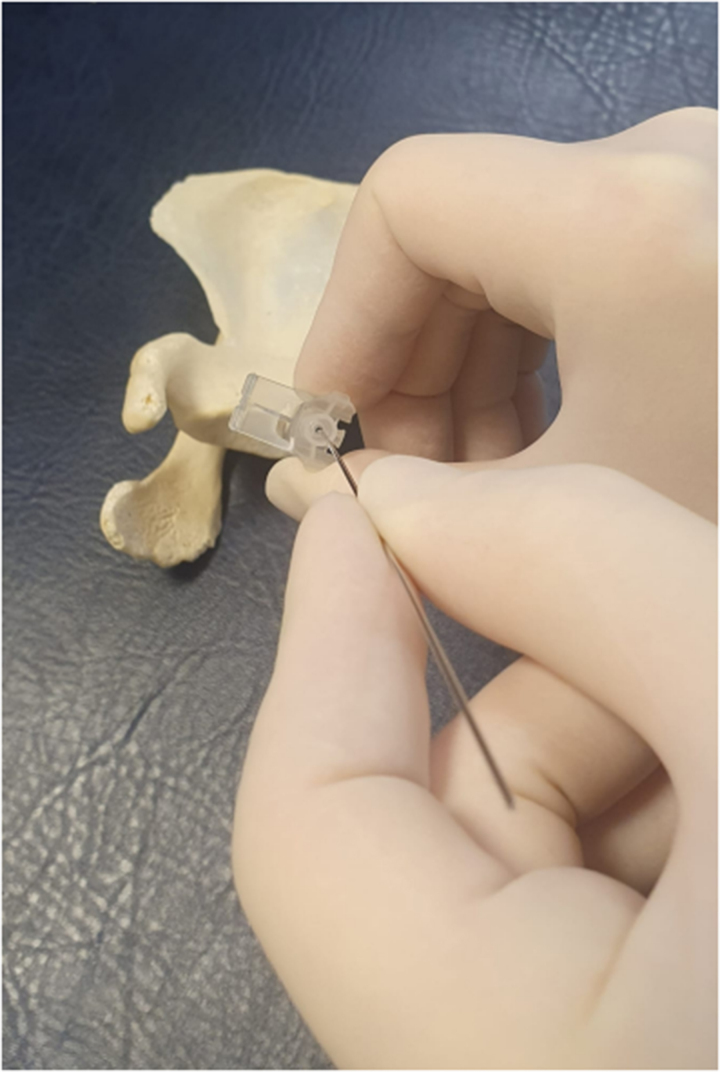
Guidewire passed down cannula

**Figure 3:**
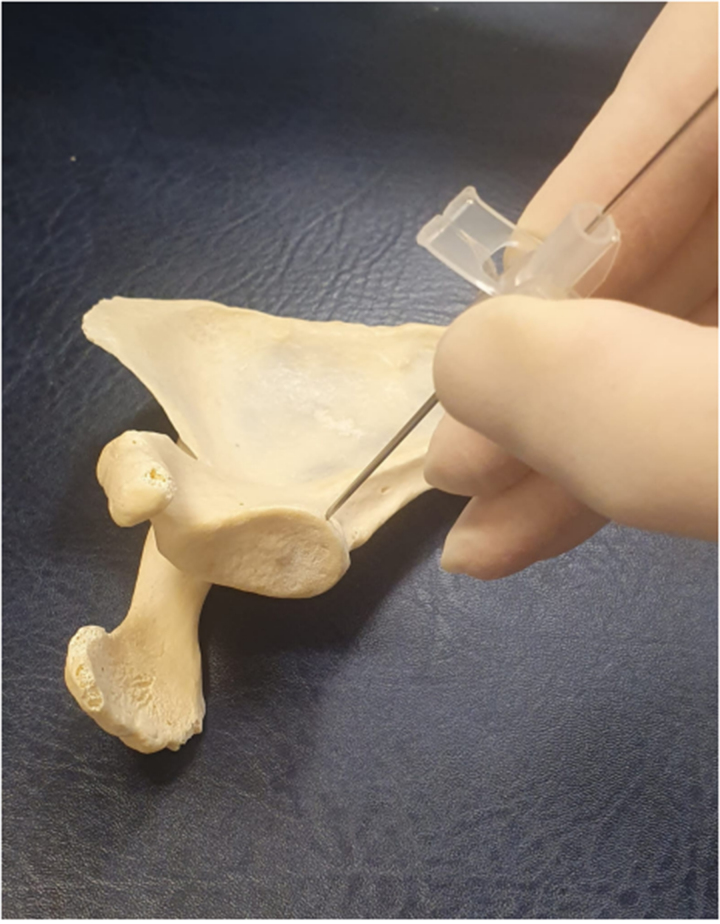
Cannula used to position and orientate the guidewire

**Figure 4:**
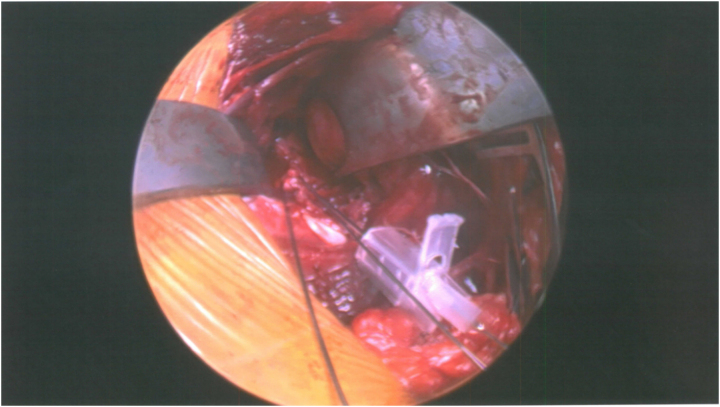
Intraoperative photograph of left glenoid fixation using cannula technique

Glenoid fracture fixation can be achieved with narrow-diameter cannulated-screw systems. This employs preliminary non-rigid guide K-wires of between 0.9 and 1.2mm in size, to ensure correct screw positioning. To aid in achieving the correct wire orientation, the wire can be passed through an orange (14-gauge) cannula needle, which has an internal diameter of 1.6mm. The cannula needle can be used to both place and orient the K-wire in the most appropriate position to achieve optimum fixation (Figures 1–4).

## Discussion

The needle point sinks into the bone and therefore is less likely to displace during passing of the K-wire. Furthermore, the cannula needle is much more rigid than the K-wire, which allows the surgeon to direct the wire with greater ease. The cannula also acts as a tissue guide, preventing soft tissue entanglement in the rotating wire. This technique we describe is particularly useful given the limited accessibility of the glenoid.
